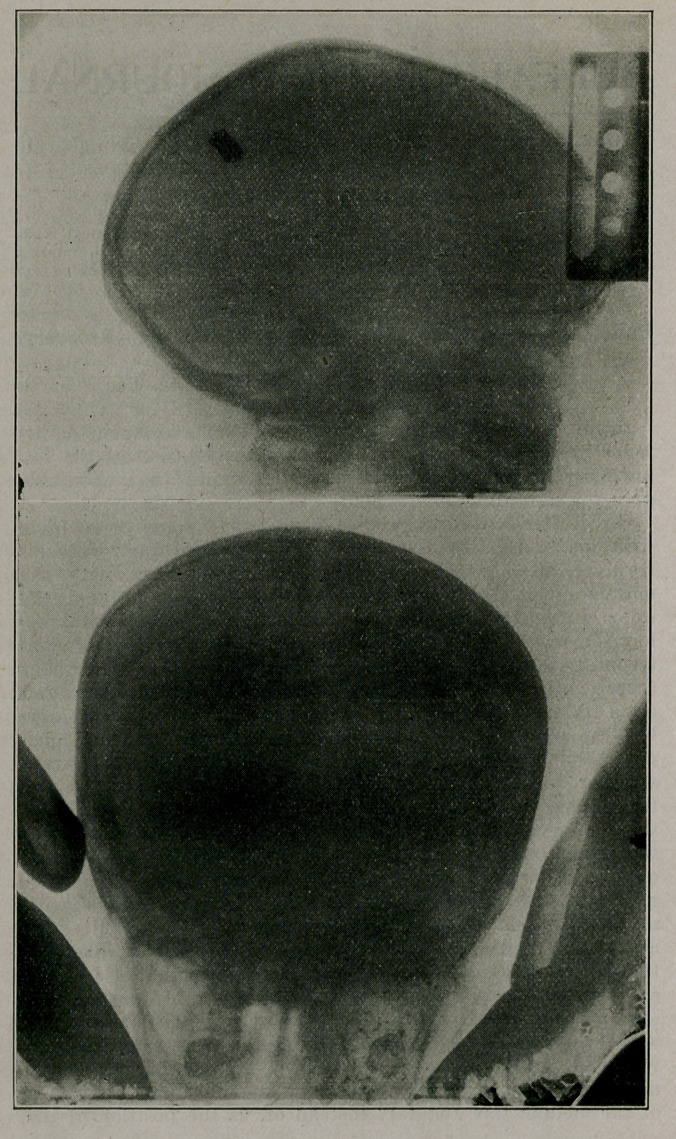# Bullet in Brain Destroying Sight and Hearing—Recovery

**Published:** 1917-05

**Authors:** Evan O’Neill Kane

**Affiliations:** Kane, Pa.


### BUFFALO MEDICAL JOURNAL

Yearly Volume 72 | MAY, 1917 | Number 10

### ORIGINAL ARTICLES

The right is reserved to decline papers not dealing with practical medical and surgical subjects, and such as might offend or fail to interest readers. Contributors are solely responsible for opinions, methods of expression and revision of proof.

---

### Bullet in Brain Destroying Sight and Hearing—Recovery.

EVAN O'NEILL KANE, M. D., Kane, Pa.

Brain injuries from projectiles are of such common occurrence in the present World War that the citation of the following case history in civil practice would be inexcusable save for its unique character.

CASE HISTORY: Clarence Irwin, aged four, in struggle with his brother of six for the possession of their father's revolver, discharged the weapon at close range with the muzzle directed towards his mouth. The ball entered the upper lip to the right of the median line one centimeter below the wing of the nose, and after traversing the brain anteroposteriorly and striking the occiput, rebounded about three centimeters and stopped turned more than half around upon its axis. Radiographs demonstrated its position clearly, but the child having been brought forty miles in an open automobile over rough country roads in severely cold winter weather, also having lost considerable blood, it was decided to postpone operation (it was 11 p. m. when he arrived) until the following morning. Trephining was not undertaken until sixteen hours after arrival. The brain tissue at the point of opening was not disorganized, though engorged and edematous. After protracted search in all directions with needles, supplemented by small spoon curette, for fully five centimeters without detecting the missile, further exploration was abandoned. A needle was pushed to the base of the explored area, packed in place with gauze, and another radiograph was taken transversely and anteroposteriorly. It was thus disclosed that through an error of the radiographer, who had placed the picture for inspection wrong side foremost, the opening had been made at a point just to the left of the median line and falx instead of, as the position of the needle in the radiograph indicated should have been done, to the right. The child was again placed upon the operating table and the same procedure repeated in the proper location. Again exploring needles were passed in all directions, but without success, despite the fact that a gush of pulpified brain tissue, blood and clots, which escaped as soon as the opening was made, proved that the missile must be in the near neighborhood. The entire area was explored more than six centimeters in every direction, save where the falx interposed a barrier. Abandoning further search in this manner, the little finger, rubber gloved, was gently inserted, and with it a more comprehensive exploration made. The bullet was then discovered well below the trephine opening, close against the cranium, where it had evidently fallen through gravity during the operation. The missile was readily removed with curved forceps; the cavity was then drained with catgut and gauze. Recovery was uneventful without fever or suppuration. Some brain hernia took place at first, but this was soon overcome by suitable pressure.

From the time of the injury until eight hours after operation the breathing continued sterterous and irregular, the pulse full, slow and variable, the pupils contracted and divergent. These ominous symptoms gradually subsided, and after six days consciousness began to develop. It became evident, however, that the little patient was both blind and deaf. He groped about helplessly at first, crying to know when daylight would come. He implored frequently that someone would bring his father's lantern from the barn, or his brother's flashlight, the wonders of which he described graphically, from the shelf where he affirmed it was hidden. He was a singularly intelligent child for his age, and showed remarkable ability in adjusting himself to the circumstances of his limited senses, in deafness and blindness playing with his toys by feel and enjoying his food by taste and smell, feeding himself quite skilfully despite a slight incoordination which made carrying food directly to the mouth somewhat uncertain. Five days later, to the joy of his attendants, it became evident that hearing, though very imperfect, was returning, and in another week it was quite clear, so that his chattered conversation with all within reach was continuous. He remained in darkness for ten more days, and had become quite cheerfully resigned to his lot when light perception began to be evidenced, and, I believe, had he remained blind would have still been happy. A week later his eyesight was excellent. At present writing, three months after accident, he is in possession of all his faculties and, save for a slight unsteadiness of gait, somewhat like that of a sailor on shore, he is entirely normal.

A line drawn from the point of entrance of the bullet to that of its arrest proves it to have traversed at first the cranial floor, and thereafter at least seven centimeters of the base of the brain scarcely two centimeters from the median line. For a missile of this size (No. 32) at close range to have taken this course it must have torn a sufficiently wide way for itself, and the structures through which it passed should have been so disorganized or thoroughly destroyed as to appear to preclude restoration. The suggestion is obvious that at this early age nerve and brain tissues have remarkable recuperative power. While fortunate from a clinical standpoint in having recovery take place, it is to be regretted that the interesting data to be derived from an autopsy are unobtainable.

230 Clay Street, Kane, Pa.

---

**Generin.** James Oliver, London, N. Y. Med. Jour., Jan. 20, claims that a special substance (hormone?) to which he gives this name is requisite for both menstruation and gestation and that women who cannot menstruate do not become pregnant. He cites 9 cases, 3 married after the usual age of the menopause but under that at which conception becomes impossible, the other six married before the age of the menopause, under observation 3 or more years and now past the menopause age. He believes that ovulation is independent of menstruation because he found Graafian follicles in an unmarried woman of 29 who had never menstruated. He distinguishes between cases that do not menstruate as on account of lactation, or pregnancy occurring soon after premature marriage and those that cannot menstruate.

---

**Pulse and Blood Pressure on the Firing Line.** Pierre Menard, Bull. de la Soc. de Med. de Paris, Oct. 27, 1916, gives tables and diagrams which he sums up as follows: In the first line, , 100-150 meters from the enemy, the maximum and minimum pressures are generally lowered, rising in the second and third lines; there is a more or less marked tachycardia in the first line. A violent emotion, as due to the fall of a shell near by, considerably raises the minimum pressure but only slightly the maximum. Fatigue and over-strain always reduce the difference between maximum and minimum tension, almost always elevating the minimum. In 2/3 of cases they increase the pulse rate, in 1/3 they reduce it.

## Figures and Tables

**Figure f1:**